# Optimisation of the Danish national haemoglobinopathy screening programme – A prospective intervention study

**DOI:** 10.1002/jha2.980

**Published:** 2024-08-08

**Authors:** Esther Agnethe Ejskjær Gravholt, Finn Stener Jørgensen, Charlotte Holm, Jesper Petersen, Amina Nardo‐Marino, Mathis Mottelson, Andreas Glenthøj

**Affiliations:** ^1^ Department of Haematology Danish Red Blood Cell Centre Copenhagen University Hospital – Rigshospitalet Copenhagen Denmark; ^2^ Department of Obstetrics and Gynaecology Fetal Medicine Unit Copenhagen University Hospital – Amager Hvidovre Hospital Hvidovre Denmark; ^3^ Department of Clinical Medicine Faculty of Health and Medical Sciences University of Copenhagen Copenhagen Denmark; ^4^ Department of Obstetrics and Gynaecology Copenhagen University Hospital – Amager Hvidovre Hospital Hvidovre Denmark

## Abstract

**Introduction:**

The Danish national haemoglobinopathy screening programme offers screening to at‐risk pregnant women. Despite efforts to increase awareness of the screening programme, most women in the target population remain unscreened. In contrast, > 90% of pregnant women in Denmark attend a screening for chromosomal abnormalities by combined first‐trimester screening (cFTS).

**Methods:**

This study aimed to improve adherence to the Danish national haemoglobinopathy screening programme by offering screening to at‐risk unscreened pregnant women in relation to their cFTS.

**Results:**

During a 27‐week intervention period, 3254 women attended cFTS at Copenhagen University Hospital—Amager Hvidovre Hospital. Of these, 938 women (28.8%) were identified as at risk of carrying haemoglobinopathy variants based on their ethnic origins. Of the 938 women at risk, 539 (57.5%) were unscreened prior to their cFTS and were targeted for the intervention. These women were contacted with an offer of haemoglobinopathy screening. Subsequently, 253/539 (46.9%) of the at‐risk unscreened women were tested for haemoglobinopathies, of these 4/253 (1.6%) carried haemoglobinopathy variants necessitating partner screening. No partners carried haemoglobinopathy variants necessitating testing of the fetus.

**Conclusion:**

The study increased the proportion of at‐risk pregnant women tested for haemoglobinopathies from 42.5% to 69.5% and made haemoglobinopathy screening more readily available to women attending cFTS.

## 1 INTRODUCTION

Haemoglobinopathies vary in frequency across the globe, with the highest prevalence in the Southeast Asia Region, the Eastern Mediterranean region and the African region [1, 2]. In Denmark, it is estimated that approximately 3%–4% of the immigrant population carry haemoglobinopathy gene variants [3, 4].

The World Health Organization recommends that all member states implement haemoglobinopathy screening programmes. Screening strategy should depend on each country's prevalence, morbidity and mortality due to haemoglobinopathies, as well as their economic capability [2]. Similarly means of testing for haemoglobinopathies varies between countries, depending on capability [5]. Haemoglobinopathy screening programmes typically follow one of three strategies: premarital, antenatal or neonatal [6]. In haemoglobinopathy‐endemic countries, screening programmes typically target the general population, whereas only at‐risk populations are targeted in non‐endemic countries [7, 8]. Premarital screening allows for the use of in vitro fertilisation and preimplantation genetic testing, and early prenatal screening provides parents with a reproductive choice. Neonatal screening ensures early diagnosis, allowing for timely treatment and thereby minimising the risk of disease‐related morbidity and mortality.

Due to an increasing prevalence of haemoglobinopathies in the Danish population, a screening programme testing for α‐ and β‐thalassaemia and major pathogenic haemoglobin variants was initiated in the Capital Region of Denmark in 1995. The screening programme was nationalised in 2007. As haemoglobinopathies remain infrequent in the Danish general population, current Danish national guidelines recommend targeted prenatal screening exclusively for women who are considered at risk of carrying a haemoglobinopathy, that is, women with ethnic origins in haemoglobinopathy‐endemic countries [4]. The aim of the screening programme is to provide parents with a reproductive choice, emphasising the importance of screening as early as possible. Hence, screening should be offered by the general practitioner (GP) pre‐conceptionally or during the first antenatal visit at gestational weeks 6–8. If couples are found to be at risk of having a child with severe haemoglobinopathy, they are referred to genetic counselling and offered prenatal testing. This screening strategy has recently been described in detail [9]. Notably, only haemoglobinopathy variants which may lead to significant illness are considered relevant for partner testing or testing of the fetus. This would encompass HbS, HbC, HbD, HbE, HbO, non‐deletional α‐variants (e.g. Hb Constant Spring), α0‐variants and β‐thalassemia variants, but exclude deletional α^+^‐thalassaemias such as the 3.7 deletion. Patients who present with microcytosis or hypochromia, are generally recommended for haemoglobinopathy testing independently of the screening programme.

**FIGURE 1:**
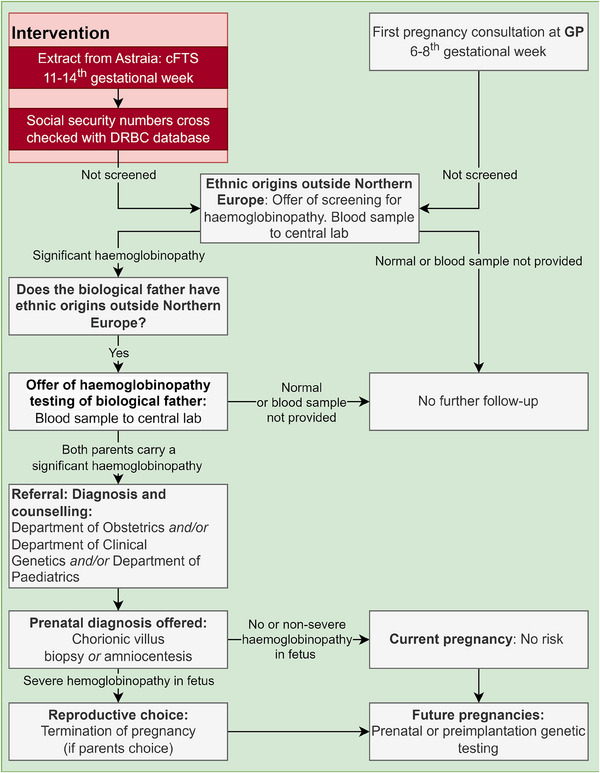
Organisation of the Danish national haemoglobinopathy screening programme and of the prospective intervention.

In Denmark, standard antenatal care is multidisciplinary involving GPs, midwives, and obstetricians, GPs are also responsible for referral for further testing of a partner or of a fetus. GPs are responsible for completing a pregnancy chart between 6 and 8 weeks of gestation. This chart compiles data on comorbidities, social factors, and other relevant information, which is then communicated to the hospital when informing them of the pregnancy. At this visit, pregnant women are also offered screening for hepatitis B, syphilis, and HIV. Subsequently, all pregnant women are offered combined first‐trimester screening (cFTS), which is performed at a hospital facility. Figure 1 illustrates the organisation of the existing Danish national haemoglobinopathy screening programme and of the intervention (the red box).

In 2009 the Danish Health Authorities conducted a sample survey in the Central Region of Denmark, which found that only 1/3 of the at‐risk population in Denmark were screened for haemoglobinopathies as advised in the Danish national guidelines [10]. Since then, no significant changes have been made to the screening programme, nor has a more recent survey been conducted. In contrast, the average national attendance rate for cFTS at the 11th–14th gestational week is estimated at 92.4% [11].

In this work, we describe a prospective intervention study aiming to increase the proportion of pregnant women at risk of carrying haemoglobinopathy genes offered and accepting screening for haemoglobinopathies. The project is a pilot study carried out in collaboration between the Danish Red Blood Cell Centre (DRBC) at Copenhagen University Hospital – Rigshospitalet, and the Fetal Medicine Unit at the Department of Obstetrics and Gynaecology at Copenhagen University Hospital—Amager Hvidovre Hospital.

## 2 METHODS

### 2.1 Design

Prospective intervention study.

### 2.2 Population

All pregnant women attending cFTS at the Department of Obstetrics and Gynaecology at Copenhagen University Hospital – Amager Hvidovre Hospital, from 2 May 2022 to 6 Nov 2022. The department is responsible for 7200 deliveries every year and the hospital's primary service area has more immigrants and descendants of immigrants than the national average.

Women who had ethnic origins outside of Northern Europe were defined as at risk of carrying haemoglobinopathies and thus, the target of the intervention.

### 2.3 Data collection

cFTS were managed by sonographers, and all data were registered in the fetal medical electronic records system, Astraia. Data from Astraia were recorded using REDCap (Research Electronic Data Capture) electronic data capture tools hosted by the Capital Region of Denmark [12, 13]. Data included name, social security number, ethnicity, gestational age, parity, estimated ultrasound‐based due date, date of cFTS and hospital department, as well as laboratory data from the DRBC database and pregnancy charts obtained from medical records.

The selected study population all resided in the Capital Region of Denmark, which is fully covered by the DRBC database, as it covers haemoglobinopathy testing in four out of five regions in Denmark (77% of the Danish population), excluding the Central Region of Denmark.

Social security numbers are unique identification numbers given to all individuals with permanent residency in Denmark and used throughout the Danish Health Care system and public sectors.

We drew data from StatBank Denmark, Statistics Denmark, to verify our target population.

### 2.4 Intervention

Data were extracted from Astraia on a weekly basis and social security numbers were cross‐checked with data from the laboratory database at the DRBC.

Women who were in the at‐risk population and who were unscreened for haemoglobinopathies prior to their cFTS were targeted for the intervention. These women were contacted by both telephone and secure electronic mail (*Digital Post*), with the offer of optional haemoglobinopathy screening by blood sampling. Only one phone call per person was attempted. It was made mandatory for all residents of Denmark to receive posts from public authorities via Digital Post in 2014, excluding those exempt for special reasons [14].

The written information sheet sent by secure electronic mail contained information about haemoglobinopathies and the organisation of the Danish national haemoglobinopathy screening programme. It also included motivation for screening. For the first 15 weeks of the intervention, the written information was only provided in Danish (*n* = 1753), with the option to request the information in English. For the remaining period of 12 weeks, information was provided in both Danish and English (*n* = 1502). Efforts were made to accommodate information in Arabic, but due to technical issues, this was unfortunately not possible. Instead, for the last period of 12 weeks, the information contained a web link to the information in an Arabic translation.

Pregnancy charts were examined for the first 15 weeks (*n* = 1753), to evaluate GPs' tendency to consider haemoglobinopathy screening and note ethnic origins.

The ethnicity of pregnant women attending cFTS was registered as per routine in Astraia as one of five categories: Caucasian, Afro‐Caribbean, East Asian, South Asian, or other. The Caucasian category included the Middle East, Southern Europe, and Northern Africa, which are all regions endemic for haemoglobinopathies. Therefore, sonographers performing cFTS were asked to ask for specifications on whether women in the Caucasian category originated outside of Northern Europe. Ethnic origin was defined as going back two generations, that is, asking women where their parents or grandparents were born or if they had known ancestry outside of Denmark. Ethnic origins were not cross‐checked in medical records. Because the DRBC database covers all haemoglobinopathy testing in the service area no women were lost to follow up. In accordance with standard practice, GPs were promptly notified, through secure e‐mail, when a pregnant woman was found to carry a haemoglobinopathy. GPs then facilitate partner testing.

### 2.5 Laboratory analyses

Laboratory analyses followed standard practice of the Danish national screening programme for haemoglobinopathies, the testing algorithm is specified in Gravholt et al. [9].

Briefly, blood samples were subject to evaluation of standard haematological parameters such as Hb, mean corpuscular volume (MCV), mean corpuscular haemoglobin (MCH) and ferritin levels as well as haemoglobinopathy testing by high‐performance liquid chromatography, using Variant II (Bio‐Rad). Gap polymerase chain reaction, testing for the most common α‐deletions(15), was only performed for samples with low MCV/MCH. To identify variants in the β‐globin gene (HBB), the samples were subjected to second‐line testing through Sanger sequencing of *HBB*, using Applied Biosystems 3500 Genetic Analyser (Life Technologies). In cases where rare deletions or Hb Constant Spring were suspected, multiplex ligation‐dependent probe amplification using SALSA MLPA Probemix (MRC Holland) was utilized. Additionally, variants in the α‐globin gene (*HBA*) were detected using Sanger sequencing [9].

## 3 RESULTS

**FIGURE 2:**
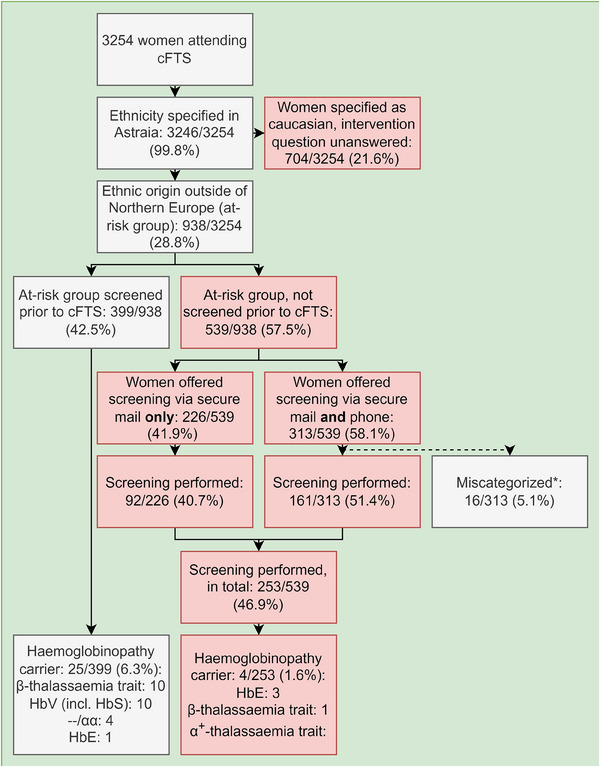
Results of the intervention, cFTS, combined first‐trimester screening. *14 women were not of relevant ethnic origins, one was pregnant via oocyte donation and one was already tested for haemoglobinopathies.

Figure 2 shows the results of the 27‐week intervention period. A total of 3254 women attended cFTS at a median of the 13th gestational week, 12 women were scanned before the 11th gestational week and five women were scanned after the 14th gestational week, which was probably due to uncertainty regarding gestational age. For 13 women gestational age was not noted. We found that 938/3254 (28.8%) of the study population had ethnic origins outside of Northern Europe, of these 539/938 (57.5%) were unscreened prior to the cFTS, and thus targeted for the intervention. These women were contacted with an offer of haemoglobinopathy screening, either via secured e‐mail only, 226/539 (41.9) or by e‐mail and phone, 313/539 (58.1%). Subsequently, 253/539 (46.9%) of the at‐risk unscreened women were tested for haemoglobinopathies, of these 4/253 (1.6%) carried haemoglobinopathy variants necessitating partner screening. 161/313 (51.4%) of the women who were successfully contacted by both phone and secure electronic mail participated in the screening programme, whilst only 92/226 (40.7%) of women who were only reached by secure electronic mail decided to participate in the screening programme.

No partners carried haemoglobinopathy variants necessitating testing of the fetus. 704/3254 (21.6%) women were categorized as Caucasian with further specification of ethnic origins lacking, thus possibly missed by the intervention.

In the pregnancy charts, GPs are asked to specify whether patients have had haemoglobinopathy testing done. Disappointingly, haemoglobinopathy screening (yes/no/deselected) was only specified for 99 out of 1753 women (5.6%) in our cohort, meaning that no decision was recorded in 1654 out of 1753 charts (94.4%). Additionally, ethnicity was only specified in 781 out of 1753 charts (44.6%).

When drawing data from StatBank Denmark, Statistics Denmark, we found that in Copenhagen University Hospital—Amager Hvidovre Hospital's primary service area, between 24%–34% of women aged 15–44 years had ethnic origins outside of Northern Europe [16].

## 4 DISCUSSION

In this prospective intervention study, we aimed to increase the proportion of at‐risk pregnant women who were offered and accepted screening for haemoglobinopathies in Denmark. Our results showed that, prior to the intervention, as few as 42.5% of at‐risk pregnant women were screened for haemoglobinopathies before their cFTS. Although this proportion is higher than previously estimated [10], adherence to the screening programme remains concerningly low. Regrettably, we cannot ascertain that all women previously tested for haemoglobinopathies were tested as part of the screening programme as this information is not provided as per routine. Women may have been tested diagnostically years before pregnancy, which the GP might not be aware of, limiting the screening value.

At present, the national Danish haemoglobinopathy screening programme relies on GPs referring women for screening during their first antenatal visit around gestational weeks 6–8. However, we found that haemoglobinopathy screening was initiated by GPs for just 5.6% of at‐risk women. Furthermore, ethnicity was noted in only 44.6% of pregnancy charts, despite the study being conducted in an area with a high prevalence of individuals of non‐Danish origin. As such, the current screening strategy is inadequate. This could be due to haemoglobinopathy screening not being a mandatory field for GPs to cross off in the pregnancy chart, clinicians not being comfortable inquiring about ethnic origins, GPs being unaware of the screening programme or simply due to time constraints in general practice. Targeted neonatal screening for sickle cell disease (SCD) based on ethnicity has previously been advised against due to several disadvantages, most importantly a high risk of failure to identify affected newborns, especially in low‐prevalence countries where clinicians may be unaware of the risk of the individual couple. Additionally, it has been argued that targeted screening can lead to stigmatisation of individuals from ethnic groups and that language barriers may pose a challenge [8]. A review evaluating the cost‐effectiveness of universal versus targeted neonatal screening for SCD in the US and UK found that even when targeted screening based on ethnicity seemed to be the most cost‐effective, universal screening was favoured due to fewer logistical and ethical challenges(17). Increasing awareness and acceptance of screening programmes in the general population via informational campaigns has proven successful in many countries such as Greece and Italy and would be worth considering in a Danish context too as national campaigns have not been implemented before [18, 19]. Another way of improving the screening programme could be drawing inspiration from the UK, where differentiated screening in the form of the implementation of universal screening in high‐prevalence areas and selective screening in low‐prevalence areas, alongside universal neonatal screening for SCD, has yielded impressive results [20, 21].

In this study, we added a safety net to the screening programme at the cFTS in the form of an offer of screening for haemoglobinopathy for previously unscreened at‐risk pregnant women. Although this intervention successfully increased adherence to the screening programme from 42.5% to 69.5% and identified several pregnant women as haemoglobinopathy carriers, only 253/539 (46.9%) of the previously unscreened women who were selected for the intervention accepted the offer of haemoglobinopathy screening. Thus, the number of screened women remained low despite our intervention. This could in part be due to technicalities such as the word “research” being denoted in the subject line of the invitation to participate in the screening programme, as opposed to “screening for haemoglobin disease” being the topic. Even so, in low‐prevalence countries like Denmark, limited public and physician awareness of haemoglobinopathies and their serious consequences could dampen motivation for screening. To address this, we included information about the diseases in layman's terms, a phone number for our haemoglobinopathy hotline, and a link to an informational webpage with the screening invitation. Efforts have been made over the years to heighten physicians’ awareness by writing articles in Danish, providing educational sessions and creating the abovementioned webpage informing of the screening programme. Another explanation could be that there is no immediate emotional benefit from participating in the screening, that is, seeing the fetus or determining the sex, which could be a driver for participation in screening by ultrasound during pregnancy. Additionally, this invitation leaves it to the pregnant woman herself to schedule an appointment for blood sampling, whereas having the blood sample taken when she is already at her GP or at the cFTS could heighten adherence to screening.

Another limitation of the current intervention, as well as the existing screening program, is its focus on identifying haemoglobinopathy carrier status during antenatal care rather than screening couples pre‐pregnancy. This issue is further emphasised as the intervention occurs in relation to cFTS which is performed at approximately the 12th gestational week, leaving little time for testing of both parents and the fetus. This timing places unnecessary emotional stress on parents, potentially confronting them with the difficult decision of late‐term pregnancy termination, which may lower the acceptance rate of pregnancy termination of an affected fetus. Investigating parents’ attitudes towards screening for haemoglobinopathies, invasive testing of a fetus and termination of pregnancy would be of great relevance in future studies, as recently addressed [9].

In our study population, 28.8% of women originated outside of Northern Europe which is in accordance with our findings from StatBank Denmark and, as expected, significantly higher than the national level (14.4% of the Danish population were immigrants or descendants of immigrants in January 2022 [22]. Being in an area with more immigrants and descendants of immigrants than the national average, we would expect higher screening rates due to GPs seeing more women to whom screening is recommended and therefore being more aware of the screening programme.

If the intervention from this study were to be implemented nationally, it would be preferable to make answering the project question in Astraia technically easier for the sonographers, as it could enhance response rates. We found that ethnicity was registered in 99% of journals from Astraia, whilst the research question regarding more detailed information on ethnicity of the Caucasian women was only answered in 78.4% of relevant cases. Unsurprisingly, a higher percentage of women chose to participate in the screening programme if they were contacted both by secured electronic mail and phone This dual approach allowed additional information to be provided and potential questions to be answered over the phone. However, contacting citizens individually via phone is highly resource‐intensive, making it impractical as a permanent solution. It is likely that there is an overlap between individuals reading secured electronic mail from the public sector, and those readily answering their phones, which could explain why more women participate in the screening programme when contacted via both means. According to a recent survey, 92% of the Danish population between 16–89 years of age report that they feel comfortable with secure electronic emails being the means of communication between them and Danish Authorities [23] meaning it should be a favourable way to reach the target group. It would be relevant to re‐evaluate whether patient information could be made shorter, easier to understand and more concise to hinder individual barriers to screening.

Based on the findings from our study, as well as an increasing number of immigrants and descendants of immigrants living in Denmark, it is worth considering whether haemoglobinopathy screening, testing for both thalassaemias and haemoglobin variants, in Denmark should be applied to all pregnancies, despite ethnic origins of the mother, preferably alongside the existing universal screening of pregnant women for hepatitis B, syphilis and HIV at 6–8. Gestational week. This would be in line with international recommendations and would relieve issues with clinicians having to identify at‐risk women, additionally, earlier screening would give parents more time to decide on invasive diagnosis of a fetus and termination of pregnancy. Furthermore, a universal screening strategy would reduce the risk of stigmatisation and, ultimately, inequality in healthcare. From an economic standpoint, screening of all pregnant women could substantially increase the costs of the screening programme. However, as the lifetime costs of haemoglobinopathies are high, a universal screening programme could be cost‐effective [9, 24].

Ultimately, as screening rates remained unsatisfactorily low despite our improved intervention, implementing this extra step in the existing screening programme does not seem favourable as it would come with a price both financially, logistically, and ethically unmatched by its effectiveness.

In conclusion, our study provided prenatal haemoglobinopathy screening to at‐risk pregnant women, increasing the proportion of at‐risk pregnant women tested for haemoglobinopathies from 42.5% to 69.5%. Efforts to better understand and minimise barriers to adherence to screening are ongoing.

## AUTHOR CONTRIBUTIONS

Esther Agnethe Ejskjær Gravholt, Finn Stener Jørgensen, Charlotte Holm, Jesper Petersen, Mathis Mottelson, Amina Nardo‐Marino and Andreas Glenthøj designed the research study and wrote the manuscript. Esther Agnethe Ejskjær Gravholt prepared the figures. All authors reviewed and edited the manuscript and approved the final version of the manuscript.

## CONFLICT OF INTEREST STATEMENT

Andreas Glenthøj has done consulting for Agios, Bristol Myers Squibb, Novartis, Novo Nordisk and Pharmacosmos, and received research support from Agois, Bristol Myers Squibb, Novo Nordisk, Saniona and Sanofi. Charlotte Holm has done consulting for Pierre Fabre and Pharmacosmos. The remaining authors declare no conflict of interest.

## ETHICS STATEMENT

The study was approved by Amager Hvidovre Hospital (D9603444). The laboratory database at the DRBC is approved by the Danish Data Protection Agency (P‐2020‐1196).

## PATIENT CONSENT STATEMENT

The authors have confirmed patient consent statement is not needed for this submission.

## CLINICAL TRIAL REGISTRATION

The authors have confirmed clinical trial registration is not needed for this submission.

## Supporting information


Supporting Information

## Data Availability

Raw data were generated at the Department of Obstetrics and Gynaecology at Copenhagen University Hospital – Amager Hvidovre Hospital and at the Danish Red Blood Cell Centre. Derived data supporting the findings of this study are available from the corresponding author (Andreas Glenthøj) on reasonable request.
