# Supplementary material for: Optimisation of the Danish national haemoglobinopathy screening programme – A prospective intervention study
Source: EJHaem. 2024 Aug 8;6(3):e980. doi: 10.1002/jha2.980 (PMC12104534; doi:10.1002/jha2.980)
Supplement: Supplementary file 1 — Supporting Information [file JHA2-6-e980-s001.docx]

**Social Media Info**

**Tweetable Summary:**

Integrating an offer for prenatal screening for haemoglobinopathies with first trimester screening improved adherence rates from 42.5% to 69.5% among at-risk pregnant women in Copenhagen, Denmark.

**Social media handles:**

@aglente

@AminaMarino
